# Stimulants Used by the Race

**Published:** 1877-04

**Authors:** 


STIMULANTS USED BY THE RACE.—It is estimated that coffee, both beans and leaves, are drank by sixty millions of the human family.

Tea of all kinds is used by five hundred millions, and opium by four hundred million; alcohol, in its various forms, by five hundred millions of the human race; tobacco is probably used by seven or eight hundred millions.

These startling facts indicate a large proportion of the race using some substances, that are either stimulants or narotics. The work of the physiologist, in the future, will be to determine the true place in nature of these substances, and indicate where their use ends, and abuse begins.—*Ex.*

